# Hsa_circ_0006948 enhances cancer progression and epithelial-mesenchymal transition through the miR-490-3p/HMGA2 axis in esophageal squamous cell carcinoma

**DOI:** 10.18632/aging.102519

**Published:** 2019-12-26

**Authors:** Zihao Pan, Jiatong Lin, Duoguang Wu, Xiaotian He, Wenjian Wang, Xueting Hu, Lei Zhang, Minghui Wang

**Affiliations:** 1 Guangdong Provincial Key Laboratory of Malignant Tumor Epigenetics and Gene Regulation, Sun Yat-Sen Memorial Hospital, Sun Yat-Sen University, Guangzhou 510120, China; 2 Department of Thoracic surgery, Sun Yat-Sen Memorial Hospital, Sun Yat-Sen University, Guangzhou 510120, China; 3 Department of Biliary-Pancreatic Surgery, The Third Affiliated Hospital, Sun Yat-sen University, Guangzhou 510630, China

**Keywords:** circRNA, esophageal squamous cell carcinoma, EMT, hsa_circ_0006948, HMGA2

## Abstract

Increasing studies have indicated that circular RNAs (circRNAs) are important in cancer progression. However, few circRNAs associated with epithelial-mesenchymal transition (EMT) have been elucidated in esophageal squamous cell carcinoma (ESCC). In this study, we aimed to identify whether hsa_circ_0006948 promotes ESCC cell EMT and explore its biological mechanisms. We first screened circRNA expression profiles using a circRNA microarray, and found that the expression of a novel circRNA, hsa_circ_0006948, is increased in 153 ESCC tissues and cell lines compared with noncancerous tissues and cell lines. Additionally, high hsa_circ_0006948 levels were positively associated with lymphatic metastasis and poor prognosis. Functionally, the assays indicated that cell proliferation, migration and invasion were promoted by hsa_circ_0006948 both in vitro and in vivo. Furthermore, we analyzed the relationship between hsa_circ_0006948 and miR-490-3p through bioinformatics, luciferase reporter assays, RNA immunoprecipitation and qRT-PCR. We found that hsa_circ_0006948 could bind directly to miR-490-3p which targets the 3’UTR of the oncogene HMGA2 to induce EMT. In conclusion, hsa_circ_0006948 was overexpressed in ESCC tissues and promoted cancer progression, and it could induce EMT by enhancing HMGA2 by sponging miR-490-3p, suggesting that hsa_circ_0006948 could be a biomarker for ESCC.

## INTRODUCTION

Esophageal cancer is the sixth leading cause of cancer deaths and the eighth most common cancers worldwide, with over 400,000 deaths annually [1, 2]. Of the histological categories, esophageal squamous cell carcinoma (ESCC) is the most common type with a poor prognosis. Although the current medical treatments have improved, the 5-year survival is still less than 16% [3]. For this reason, it is important to better understand the molecular mechanism of ESCC progression and to identify the ideal prognosis biomarkers of ESCC.

As a novel non-coding RNA, circular RNA (circRNA) regulates eukaryote gene expression [4, 5]. CircRNAs are formed by back-splicing covalently joined 3′ - and 5′ -ends [6], which is unlike the canonical splicing of linear RNAs. During the past decades, circRNAs have been identified as nonfunctional byproducts. In recent years, with the development of high-throughput sequencing analysis, many exonic and intronic circRNAs have been identified across the eukaryotic lineage, and the data have indicated that circRNAs are not simply byproducts of splicing errors and that they may have regulatory roles [7]. Additionally, previous studies indicated that circRNAs have important functions in carcinogenesis and showed diagnostic value [8, 9], suggesting that circRNAs may function as microRNA (miRNA) sponges to regulate gene expression. For example, circHIPK3 acts as a miRNA sponge to suppress cell proliferation in human cancers [10, 11]. Han found that circMTO1 is a sponge of miR-9 that suppresses hepatocellular carcinoma progression [12]. To date, several circRNAs have been reported in ESCC [13–15], however, few studies have elucidated the functions and underlying mechanism of certain circRNAs in the epithelial-mesenchymal transition (EMT) process of ESCC.

In this study, using a circRNA microarray profiling, we identified that hsa_circ_0006948, which originates from exons 2, 3 and 4 of the FNDC3B gene, was up-regulated in ESCC tissues and cell lines. Next we found that high expression of hsa_circ_0006948 was associated with lymphatic metastasis and poor prognosis. Further studies suggested that hsa_circ_0006948 promoted proliferation, migration and invasion, and induced EMT in ESCC cells by sponging miR-490-3p. In summary, this study indicated that hsa_circ_0006948 may play an important regulatory role in the EMT process of ESCC cells.

## RESULTS

### Expression profiles of circRNAs and characterization of hsa_circ_0006948 in ESCC

**Figure 1:**
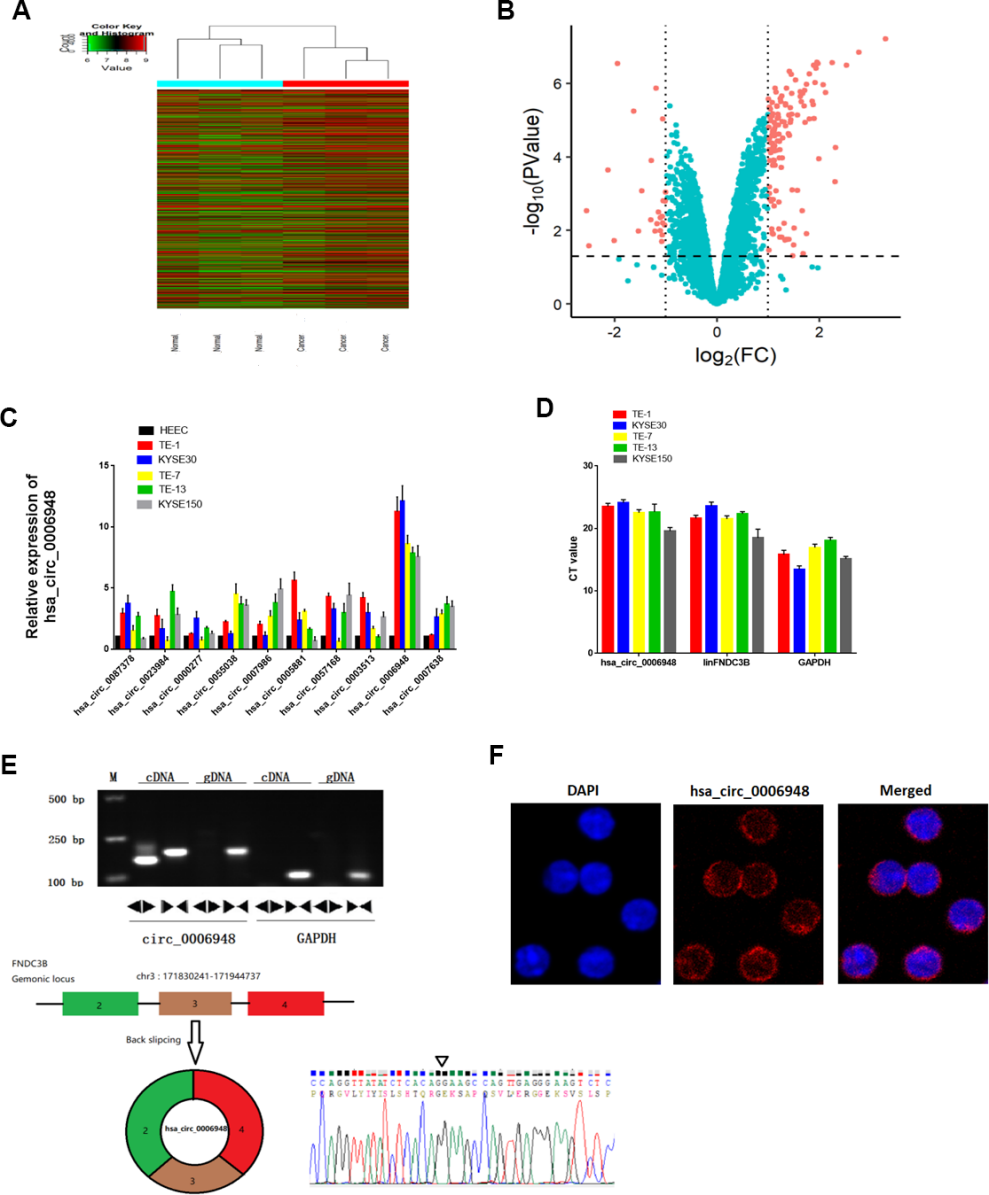
**The identification and characteristics of hsa_circ_0006948 in ESCC cells.** (**A**) Heat map showing the differential expression and hierarchical clustering of circRNAs between ESCC and adjacent normal tissues. (**B**) Volcano plot, x-axis: log2 (fold change); y-axis: -log10 (P-value). The vertical lines correspond to 2.0-fold up and down, and the horizontal line represents a P-value of 0.05. The red points in the plot represent differentially expressed circRNAs with statistical significance. (**C**) The relative hsa_circ_0006948 was significantly high in ESCC cells. (**D**) q RT-PCR analyses of expression of hsa_circ_0006948, linFNDC3B and GAPDH in various ESCC cell lines. Y-axis is the raw CT value. (**E**) Above: Divergent primers detected circular RNAs in cDNA but not gDNA. Below: Three exons form hsa_circ_0006948 by back splicing from chromosomal region and Sanger sequencing of hsa_circ_0006948 showed the back-splice junction (∇). (**F**) Fluorescence in situ hybridization assay was conducted to determine the subcellular localization of hsa_circ_0006948.

Briefly, the circRNA expression profiles of three paired ESCC tissue samples were analyzed using a microarray we deposited at Gene Expression Omnibus previously. Distinct circRNA expression profiles were shown in the hierarchical clustering (Figure 1A). A volcano plot indicated differential expression between tumor and normal tissues (Figure 1B). A significant difference was defined as a fold change > 2.0 and P < 0.05. We examined the 10 most upregulated circRNAs using qRT-PCR and found that hsa_circ_0006948 expression was significantly high in five ESCC cell lines normalized to HEEC cells (Figure 1C). Therefore, hsa_circ_0006948 was further studied. Next, we tried to investigate the relative abundance of hsa_circ_0006948 compared to its cognate linear RNA. The results showed that hsa_circ_0006948 was significantly lower than housekeeping mRNA GAPDH, however, it was abundant as the linear FNDC3B (linFNDC3B) (Figure 1D). It has been reported that the ratio between most circRNAs and their linear counterpart is about 1% [16]. Given the expression of hsa_circ_0006948 in comparison with linFNDC3B, we speculated that hsa_circ_0006948 is functional in ESCC cell lines.

Two sets of primers were designed. Divergent primers were used to amplify only the circular form, hsa_circ_0006948, while convergent primers were used to amplify only the linear form, FNDC3B mRNA. The PCR products were validated by electrophoresis using cDNA and genomic DNA (gDNA) as templates. The results revealed that the single product of the expected size was amplified distinctly, with the divergent primers for cDNA but not gDNA (Figure 1E). The Sanger sequencing results further confirmed head-to-tail splicing. These results indicated that hsa_circ_0006948 was derived from exon 2, exon 3 and exon 4 of the FNDC3B gene, which was consistent with the hsa_circ_0006948 data from circBase (Figure 1E). Further fluorescence in situ hybridization (FISH) indicated that hsa_circ_0006948 was localized preferentially in the cytoplasm (Figure 1F).

### Hsa_circ_0006948 is upregulated in ESCC and related to poor survival

**Figure 2:**
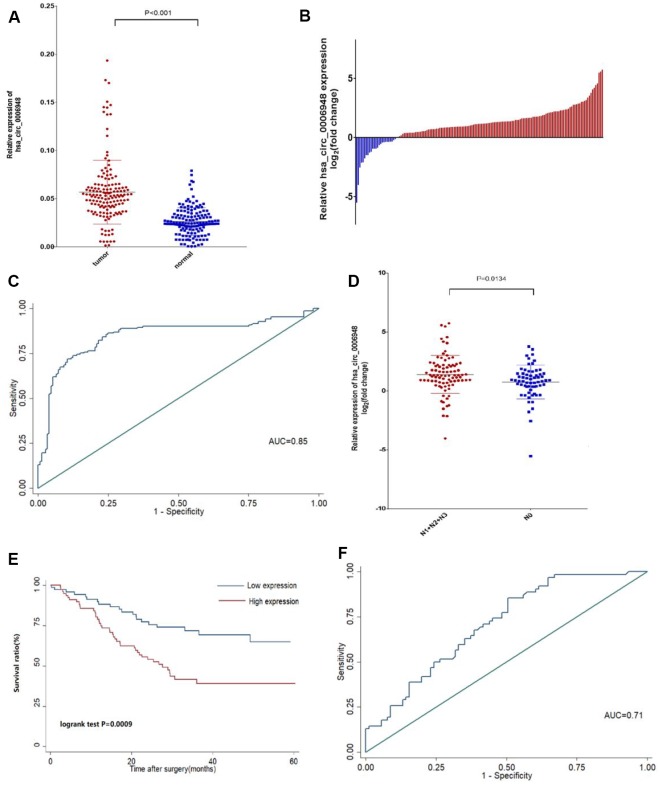
**The hsa_circ_0006948 was up-regulated in ESCC tissues and correlates with poor patients prognosis.** (**A**) hsa_circ_0006948 expression was assessed in cancer tissues and normal tissues. (**B**) The relative hsa_circ_0006948 expression was assessed in 153 ESCC patients. Blue: low level: Red: high level. (**C**) Present the ROC curve analysis of hsa_circ_0006948 for the diagnosis of ESCC. (**D**) The expression of hsa_circ_0006948 was significantly higher in patients with lymph nodes at N1-N3 stage than in those with lymph nodes at NO stage. (**E**) Kaplan-Meier’s analyses of correlations between the hsa_circ_0006948 expression levels and OS (overall survival) of 153 ESCC patients. (**F**) Present the ROC curve analysis of hsa_circ_0006948 for the prognosis of ESCC.

We examined hsa_circ_0006948 expression in 153 pairs of ESCC tissues and adjacent normal tissues using qRT-PCR analysis and found that hsa_circ_0006948 was significantly higher in cancer tissues than in normal tissues (Figure 2A). Additionally, hsa_circ_0006948 expression was high in 83.6% of ESCC patients (128 of 153) (Figure 2B). We further analyzed ROC of hsa_circ_0006948 expression to investigate its diagnostic value and found that hsa_circ_0006948 level could differentiate ESCC from normal control, with AUC of 0.85 (cutoff = 0.415, sensitivity = 0.74, specificity = 0.88) (Figure 2C). Then, the relationship between hsa_circ_0006948 and clinicopathological features was analyzed after patients were classified into high and low level groups via the median hsa_circ_0006948 expression (Table 1). Briefly, high hsa_circ_0006948 was positively correlated with lymphatic metastasis (Figure 2D), and no significant correlation was observed for other clinicopathological features, including age, T stage, sex, blood type, tumor location and differentiation. Kaplan–Meier survival curves showed that patients with higher hsa_circ_0006948 expression had a significantly shorter OS than patients with low expression (P=0.0009) (Figure 2E). To assess the prognosis value of hsa_circ_0006948, survival ROC was performed. As shown in Figure 2F, the AUC for hsa_circ_0006948 in prediction of OS was 0.71 (cutoff = 3.71, sensitivity = 0.39, specificity = 0.85). Furthermore, univariate and multivariate COX analyses were performed (Table 2). We found that the expression of hsa_circ_0006948 was an independent prognostic factor for ESCC prognosis (HR = 1.94; 95% CI 1.11-3.39; P = 0.021).

**Table 1:** Relationship between clinicopathological characteristics and hsa_circ_0006948 expression in ESCC patients.

**Characteristics**	**NO.(%)**	**hsa_circ_0006948 expression**		
		**Low(%)**	**High(%)**	**P-value**
Sex				
Male	120(78.43)	58(48.33)	62(51.67)	0.527
Female	33(21.57)	18(54.55)	15(45.45)	
T stage				
T1	23(15.03)	12(52.17)	11(47.83)	0.954
T2	21(13.73)	10(47.62)	11(52.38)	
T3	109(71.24)	54(49.54)	55(50.46)	
N stage				
N0	64(41.83)	39(60.94)	25(39.06)	0.018^*^
N1+N2+N3	89(58.17)	37(41.57)	52(58.43)	
Location				
Upper thoracic	25(16.34)	11(44.00)	14(56.00)	0.714
Middle thoracic	99(64.71)	49(49.49)	50(50.51)	
Low thoracic	29(18.95)	16(55.17)	13(44.83)	
Blood Type				
A	42(27.45)	17(40.48)	25(59.52)	0.509
B	38(24.84)	19(50.00)	19(50.00)	
AB	12(7.84)	6(50.00)	6(50.00)	
O	61(39.87)	34(55.74)	27(44.26)	
Differentiation				
Poor	47(30.72)	19(40.43)	28(59.57)	0.267
Moderate	75(49.02)	39(52.00)	36(48.00)	
Well	31(20.26)	18(58.06)	13(41.94)	
Age(years, mean±SD)		61.05±8.36	62.12±7.55	0.404

*P<0.05.

**Table 2:** Univariate and multivariate Cox-regression analysis of prognostic factors for ESCC patients (n=153).

	**Univariate analysis**				**Multivariate analysis**		
	**HR**	**95% CI**	**P**		**HR**	**95% CI**	**P**
T stage							
T1	1				1		
T2	3.59	(0.95,13.55)	0.059		2.87	(0.75,11.04)	0.124
T3	4.96	(1.55,15.89)	0.007^**^		3.99	(1.22,13.08)	0.022^*^
N stage							
N0	1				1		
N1+N2+N3	2.64	(1.49,4.67)	0.001^**^		1.88	(1.04,3.39)	0.037^*^
Sex							
Male	1				1		
Female	0.26	(0.10,0.65)	0.004^**^		0.23	(0.90,0.59)	0.002^**^
Location							
Upper thoracic	1				-		
Middle thoracic	0.94	(0.48,1.85)	0.869		-	-	-
Low thoracic	1.09	(0.49,2.43)	0.834		-	-	-
Blood Type							
A	1				-		
B	0.47	(0.22,1.00)	0.051		-	-	-
AB	1.37	(0.60,3.12)	0.604		-	-	-
O	0.79	(0.44,1.43)	0.440		-	-	-
Differentiation							
Poor	1				1		
Moderate	1.12	(0.65,1.93)	0.677		0.86	(0.49,1.51)	0.598
Well	0.33	(0.13,0.81)	0.016^*^		0.28	(0.11,0.72)	0.008^**^
Expression of hsa_circ_0006948							
Low	1				1		
High	2.40	(1.41,4.11)	0.001^**^		1.94	(1.11,3.39)	0.021^*^

*P<0.05.

**P<0.01.

***P<0.001.

### Hsa_circ_0006948 promotes the proliferation, migration and invasion of ESCC cells

**Figure 3:**
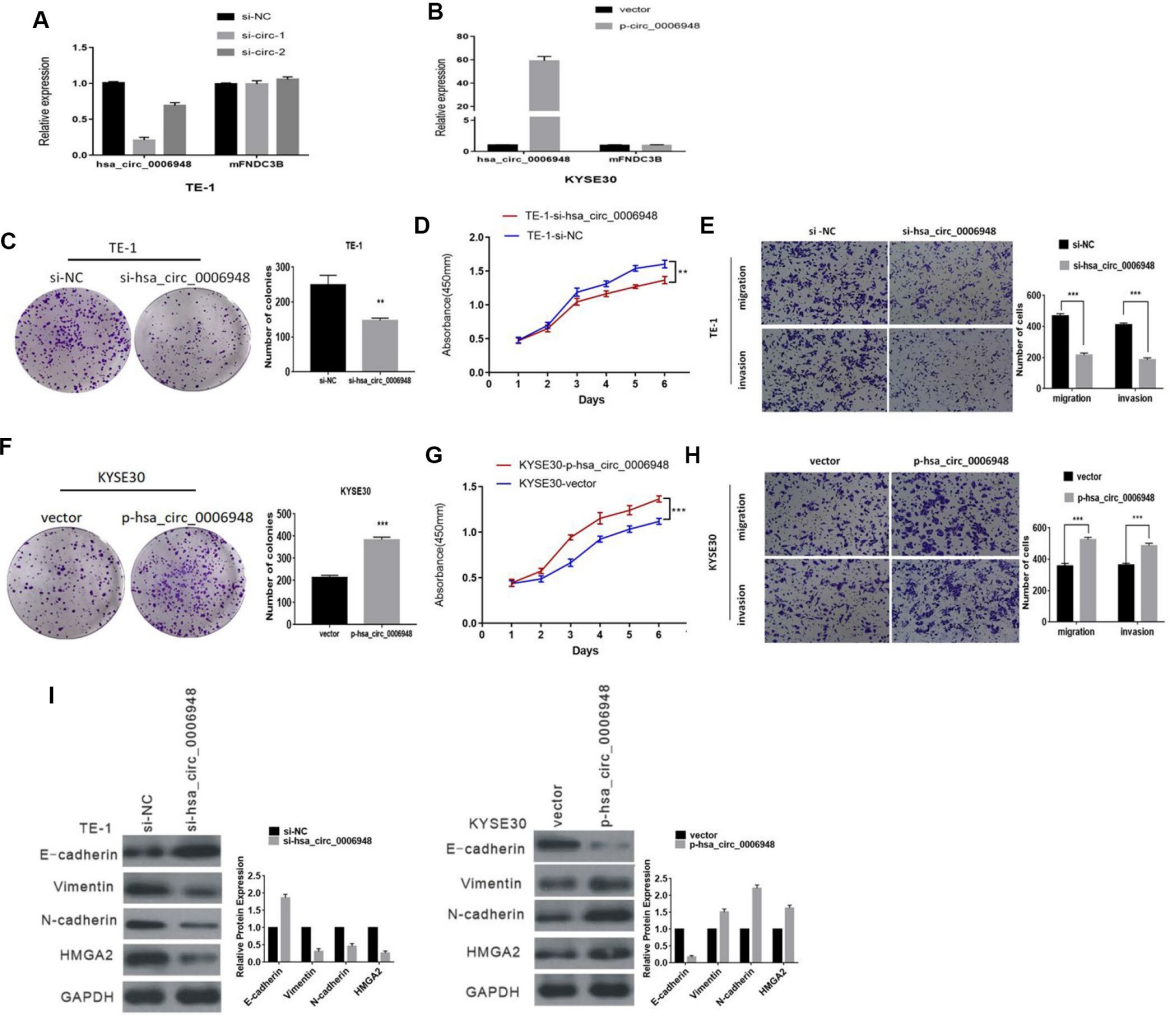
**The function of hsa_circ_0006948 in ESCC cells.** (**A**) Expression of hsa_circ_0006948 and FNDC3B mRNA in TE-1 cells transfected with siRNAs. and (**B**) KYSE30 cells overexpressing hsa_circ_0006948. (**C** and **D**) The effect of hsa_circ_0006948 on cell proliferation in vitro using colony formation assay and CCK8 assay after knocking down hsa_circ_0006948 in TE-1. (**E**) Cell migration and invasion abilities were assessed by transwell assay after knocking down hsa_circ_0006948 in TE-1 cells. (**F** and **G**) The effect of hsa_circ_0006948 on cell proliferation in vitro using colony formation assay and CCK8 assay after overexpressing hsa_circ_0006948 in KYSE30 cells. (**H**) Cell migration and invasion abilities were assessed by transwell assay after overexpressing hsa_circ_0006948 in KYSE30 cells. (**I**) Western blot analysis comparing downregulated and upregulated-hsa_circ_0006948 ESCC cells with control cells were shown for vimentin, E-cadherin and N-cadherin* P<0.05,**P<0.01, ***P<0.001.

After transfection with siRNAs (siRNA1 and siRNA2) targeting the back-splice region, hsa_circ_0006948 expression in TE-1 and KYSE30 cells was knocked down, and there was no significant change in its linear counterpart, FNDC3B mRNA (Figure 3A, Additional file 1: Supplementary Figure 1A). However, siRNA1 was more effective than siRNA2 in the knockdown of hsa_circ_0006948 expression. Thus, siRNA1 was selected for subsequent studies. Additionally, the hsa_circ_0006948 overexpression plasmid was constructed successfully, and did not affect FNDC3B mRNA (Figure 3B, Additional file 1: Supplementary Figure 1B). Colony formation assays proved that the clonogenic ability of ESCC cells was inhibited by hsa_circ_0006948 downregulation (Figure 3C, Additional file 1: Supplementary Figure 1C). To investigate the functions of hsa_circ_0006948 in cellular proliferation, CCK8 assays were performed. Compared to the negative control conditions, knockdown of hsa_circ_0006948 significantly inhibited ESCC cell proliferation (Figure 3D, Additional file 1: Supplementary Figure 1D). Then, transwell assays were performed using ESCC cells transfected with siRNA.

The transwell assay results showed that hsa_circ_0006948 downregulation inhibited ESCC cell migratory and invasion abilities. (Figure 3E, Additional file 1: Supplementary Figure 1E). In contrast, hsa_circ_0006948 upregulation yielded the reverse effects. Overexpression of hsa_circ_0006948 in ESCC cells promoted the proliferation and increased the migratory and invasion abilities in ESCC cells (Figure 3F–H, Additional file 1: Supplementary Figure 1F–1H).

### Hsa_circ_0006948 induces ESCC cell EMT

Epithelial-mesenchymal transition (EMT) is an important process in tumor aggressiveness. Therefore, we assessed whether hsa_circ_0006948 could induce EMT in ESCC cells, and EMT markers were assessed by Western blot in the present study. Western blot analysis demonstrated that knockdown of hsa_circ_0006948 led to the upregulated expression of the epithelial marker E-cadherin and downregulated expression of the mesenchymal markers vimentin and N-cadherin. On the other hand, the expression of E-cadherin was decreased, whereas that of vimentin and N-cadherin was increased in ESCC cells overexpressing hsa_circ_0006948 (Figure 3I). These results implied that hsa_circ_0006948 promotes ESCC cell EMT.

### Hsa_circ_0006948 functions as a miR-490-3p sponge in ESCC cells

**Figure 4:**
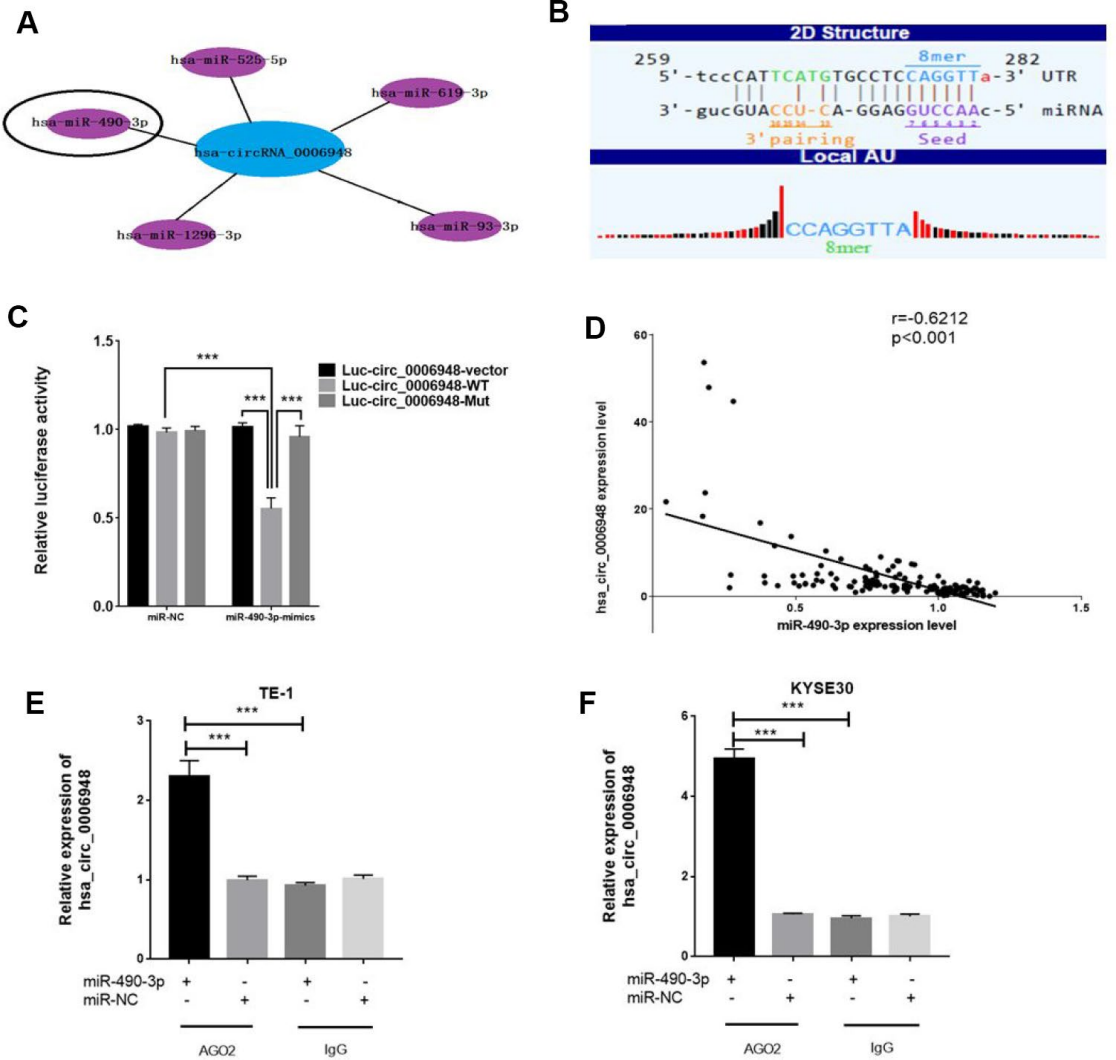
**Hsa_circ_0006948 could serve as a miR-490-3p sponge in ESCC cells.** (**A**) Top five miRNA targets are displayed. (**B**) A schematic drawing showing the putative binding sites of the miR-490-3p associated with hsa_circ_0006948. (**C**) Luciferase reporter assay for the luciferase activity of Luc-circ_0006948 WT or Luc-circ_0006948 mutant in cells cotransfected with miRNA mimics. Data are the mean±SD of three experiments. (**D**) Hsa_ciic_0006948 expression was negatively correlated with miR-490-3p expression in 153 patients with ESCC. (**E** and **F**) RNA immunoprecipitation (RIP) assays were performed using an anti-AGO2 antibody with the transfection of miR-490-3p mimics (miR-490-3p) or miR-NC in TE-1 and KYSE30 cells to detect hsa_circ_0006948 expression according to qRT-PCR. ***P<0.001.

For circRNAs, acting as miRNA sponges is the most commonly reported function pattern, and circRNAs in the cytoplasm may act as completing endogenous RNAs to bind miRNAs [17, 18]. Given that FISH showed that hsa_circ_0006948 was localized mainly in the cytoplasm, we speculated that hsa_circ_0006948 could sponge miRNAs to inhibit their function. Therefore, we tried to identify candidate miRNAs that may sponge hsa_circ_0006948. The top five miRNA targets are displayed according to bioinformatics analysis (Figure 4A). One of these five miRNAs, miR-490-3p was reported to inhibit EMT in ESCC cells by targeting HMGA2 in a previous ESCC study[19], and the molecular interaction between hsa_circ_0006948 and miR-490-3p is shown in Figure 4B. Therefore, we hypothesized that hsa_circ_0006948 could induce EMT by enhancing HMGA2 by sponging miR-490-3p. To identify whether miR-490-3p can bind to hsa_circ_0006948, a luciferase reporter assay was performed. We found that the luciferase reporter activity was reduced by nearly 50% in only cells co-transfected with miR-490-3p mimics and luc-circ_0006948-wild type containing the complete hsa_circ_0006948 sequence, and overexpression of miR-490-3p did not affect the luciferase activity of the vector with the mutant miR-490-3p binding site in cells (Figure 4C). In addition, hsa_circ_0006948 expression was negatively correlated with miR-490-3p expression in 153 ESCC patients according to q RT-PCR (r=-0.6212, p<0.001) (Figure 4D). Furthermore, we performed RIP assay for AGO2 in TE-1 and KYSE30 cells. The results of qRT-PCR showed that hsa_circ_0006948 was significantly enriched in AGO2-containing beads samples compared to control IgG immunoprecipitates, suggesting miR-490-3p targets directly hsa_circ_0006948 in an AGO2-dependent manner (Figure 4E, 4F). These results indicated that hsa_circ_0006948 may function as a competitive endogenous RNA by sponging miR-490-3p.

### miR-490-3p functions as a tumor suppressor and targets HMGA2 in ESCC

**Figure 5:**
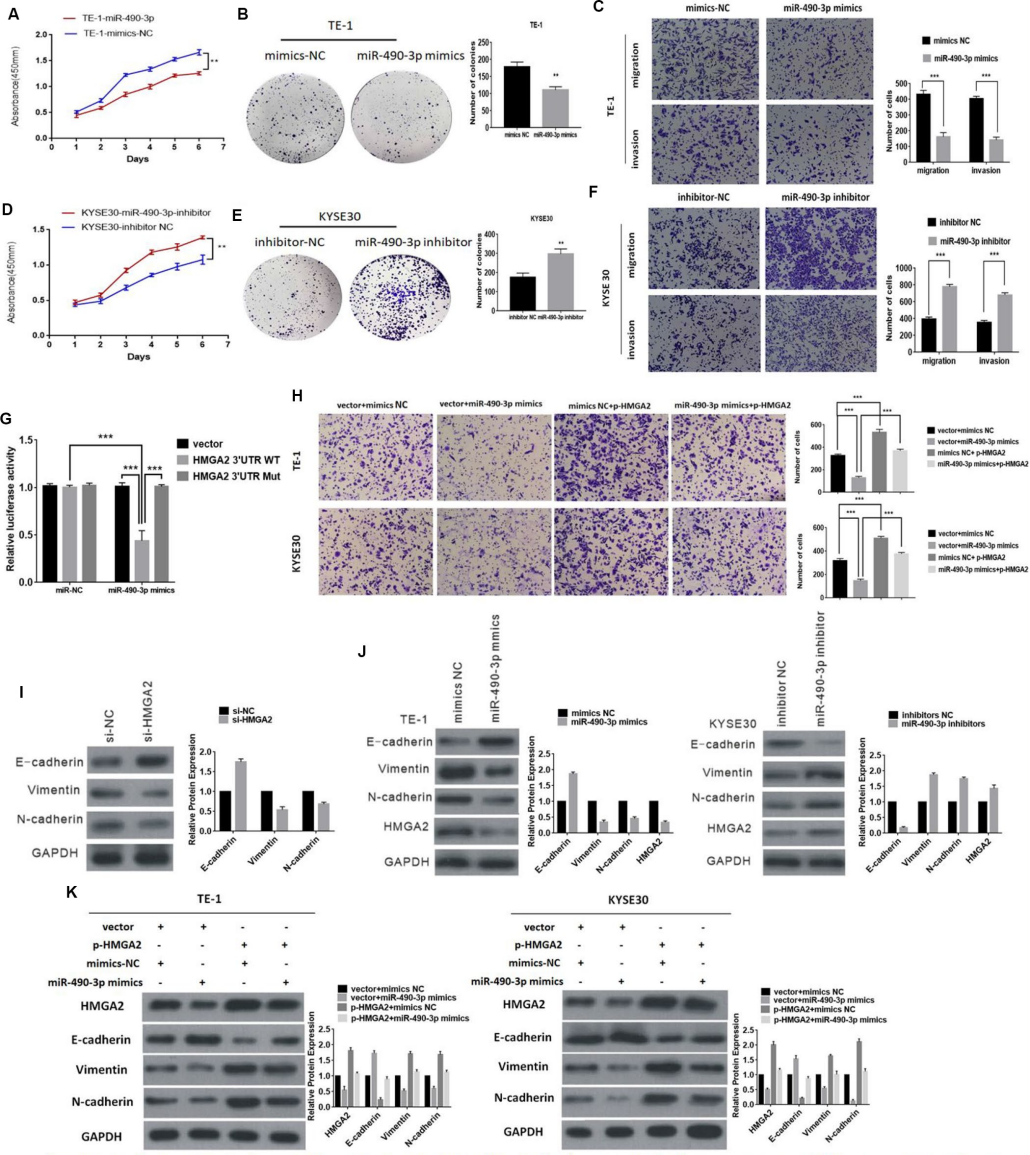
**The function of miR-490-3p in ESCC cells.** (**A**) and (**B**) The effect of miR-490-3p on cell proliferation in vitro using CCK8 assay and colony formation assay after overexpressing miR-490-3p in TE-1. (**C**) Cell migration and invasion abilities were assessed by transwell assay after overexpressing miR-490-3p in TE-1 cells. (**D** and **E**) The effect of miR-490-3p on cell proliferation in vitro using CCK8 assay and colony formation assay after knocking down miR-490-3p in KYSE30 cells. (**F**) Cell migration and invasion abilities were assessed by transwell assay after knocking down miR-490-3p in KYSE30 cells. (**G**) Luciferase reporter assay for the luciferase activity of HMGA2-3 ′UTR WT or HMGA2-3 ’UTR mutant in cells cotransfected with miRNA mimics. (**H**) The invasion ability was evaluated by transwell Matrigel invasion assays (**I**) Knockdown of HMGA2 inhibits EMT (**J**) Western blot analysis comparing upregulated and downregulated- miR-490-3p ESCC cells with control cells were shown for vimentin, E-cadherin, N-cadherin and HMGA2. (**K**) The expression of HMGA2 and EMT markers was detected by Western blot after transfection with mimics or HMGA2 overexpression plasmids.* P<0.05,**P<0.01, **P<0.001.

A previous study has verified that miR-490-3p inhibited the proliferation and metastasis of ESCC through experiments using miR-490-3p upregulation in ESCC cells [19]. To further confirm its role as a tumor suppressor, loss-of-function and gain-of-function assays were performed. Briefly, proliferative, migratory and invasive abilities were significantly inhibited after ESCC cells were transfected with miR-490-3p mimics (Figure 5A–5C). When the cells were transfected with a miR-490-3p inhibitor, the opposite results were observed (Figure 5D–5F). Additionally, previous study illustrated that HMGA2 was shown to be a direct target of miR-490-3p and induces EMT [19]. Therefore, 3’UTR of HMGA2 were constructed and inserted into vector. The result showed that luciferase activity decreased after cotransfection of miR-490-3p mimics with HMGA2-3′UTR compared with mutant vector, (Figure 5G) suggesting that miR-490-3p could bind to the 3′UTR of HMGA2.

HMGA2 have been reported to promote EMT, which was further verified in this study (Figure 5I). Additionally, miR-490-3p could regulate the expression of HMGA2 and EMT biomarkers (Figure 5J). Our further results indicated that the invasion ability inhibited by miR-490-3p was abolished by HMGA2 overexpression (Figure 5H). Similarly, upregulated miR-490-3p led to decreased expression levels of N-cadherin and vimentin, and this effect was reversed by HMGA2 (Figure 5K). Together, these results indicated that miR-490-3p inhibits EMT of ESCC by targeting HMGA2.

### Hsa_circ_0006948 upregulates HMGA2 and induces EMT by sponging miR-490-3p

**Figure 6:**
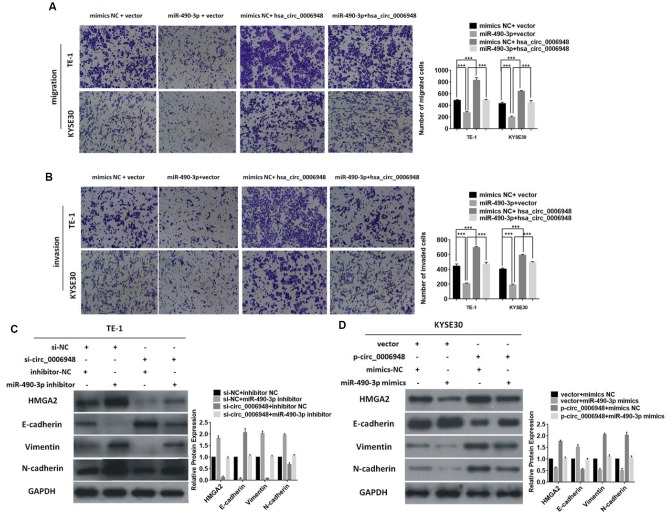
**Overexpression of hsa_circ_0006948 reverses the suppressive roles of miR-490-3p in ESCC.** The migration (**A**) and invasion (**B**) abilities inhibited by miR-490-3p were reversed after co-transfection with hsa_circ_0006948 using transwell assay. (**C**) The expression of levels of vimentin, N-cadherin and HMGA2 were inhibited by down-regulated hsa_circ_0006948, but this effect was reversed by miR-490-3p inhibitor presented in Western blot analysis. (**D**) The expression of levels of vimentin, N-cadherin and HMGA2 were enhanced by up-regulated hsa_circ_0006948, but this effect was reversed by miR-490-3p mimics presented in Western blot analysis. * P<0.05, **P<0.01, ***P<0.001.

Next, we tried to analyze whether hsa_circ_0006948 exerts its cancerous effect on ESCC by sponging miR-490-3p, and a “rescue” experiment was performed to assess the functional interaction of “hsa_circ_0006948/miR-490-3p”. Compared with the migratory and invasive abilities of ESCC cells transfected with empty vector and miR-490-3p mimics, those of upregulated hsa_circ_0006948 cells transfected with miR-490-3p mimics significantly increased, suggesting that hsa_circ0006948 promotes ESCC progression and partly reverses the tumor suppressive effect of miR-490-3p (Figure 6A 6B). Further Western blot analysis indicated that knockdown of hsa_circ_0006948 could induce downregulation of HMGA2, vimentin and N-cadherin while increasing E-cadherin. Additionally, the above phenomenon was partially restored after treatment with a miR-490-3p inhibitor in downregulated hsa_circ_0006948 ECSS cells (Figure 6C). In contrast, the opposite effect on the expression of HMGA2, E-cadherin, vimentin, and N-cadherin was observed when miR-490-3p mimics were coinfected in hsa_circ_0006948 overexpressing cells (Figure 6D). Accordingly, the result revealed that hsa_circ_0006948 might act as a miR-490-3p sponge to regulate HMGA2 expression and induces EMT.

### Silencing of HMGA2 reverses the oncogenic effect induced by upregulation of hsa_circ_0006948

**Figure 7:**
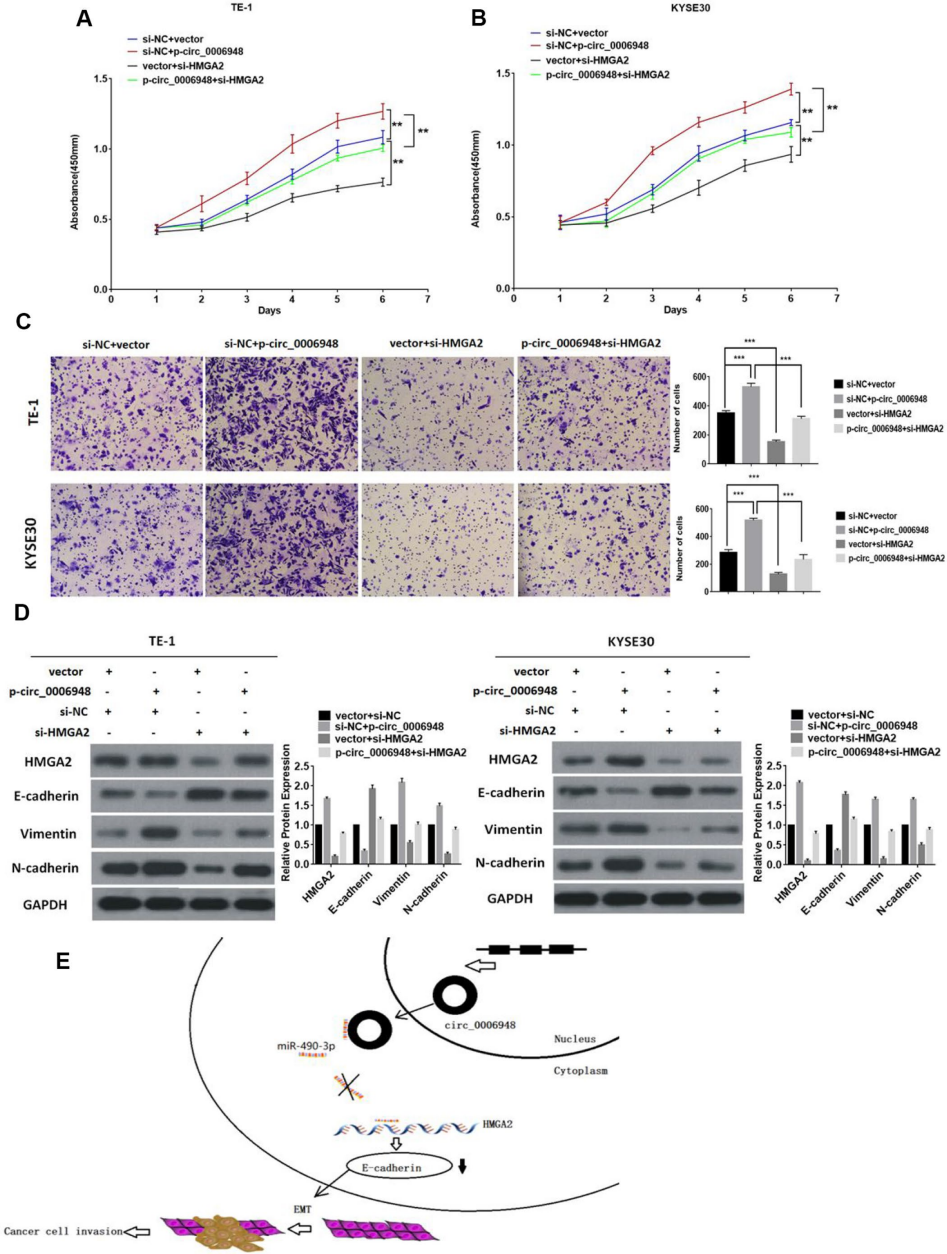
**Knockdown of HMGA2 abolishes the oncogenic effect induced by hsa_circ_0006948 in ESCC.** (**A** and **B**) The cell proliferation was measured by CCK8 assays. (**C**) The invasion ability was evaluated by transwell Matrigel invasion assays. (**D**) The upregulation of vimentin, N-cadherin. HMGA2 and the downregulation of E-cadherin in TE-1 and KYSE30 cells transfected with hsa_ciic_0006948 overexpression plasmid were abolished by knockdown of HMGA2 as detected by Western blot analysis. (**E**) A mechanistic model: hsa_circ_0006948 functions as a miR-490-3p sponge and regulates HMGA2 through inhibiting miR-490-3p activity in ESCC cells' EMT. *P<0.05, **P<0.01, ***P<0.001.

To investigate whether hsa_circ_0006948 could promote cancer progression and induce EMT by enhancing HMGA2, we transfected ESCC cells with HMGA2 siRNA and hsa_circ_0006948 overexpression plasmid. Cell proliferation assay indicated that hsa_circ_0006948 promoted cell viability of ESCC cells, while downregulation of HMGA2 reversed this effect (Figure 7A, 7B). Additionally, transwell invasion assays showed that cell invasion ability was enhanced by hsa_circ_0006948, but was restored by transfection of si-HMGA2 (Figure 7C). Moreover, knockdown of HMGA2 abrogated hsa_circ_0006948-induced increase on HMGA2, vimentin and N-cadherin, and decrease on E-cadherin (Figure 7D). Collectively, these results illustrated that HMGA2 plays a vital role in the hsa_circ_0006948/HMGA2/EMT aixs (Figure 7E).

### Hsa_circ_0006948-miR-490-3p affects ESCC growth and proliferation in vivo

**Figure 8:**
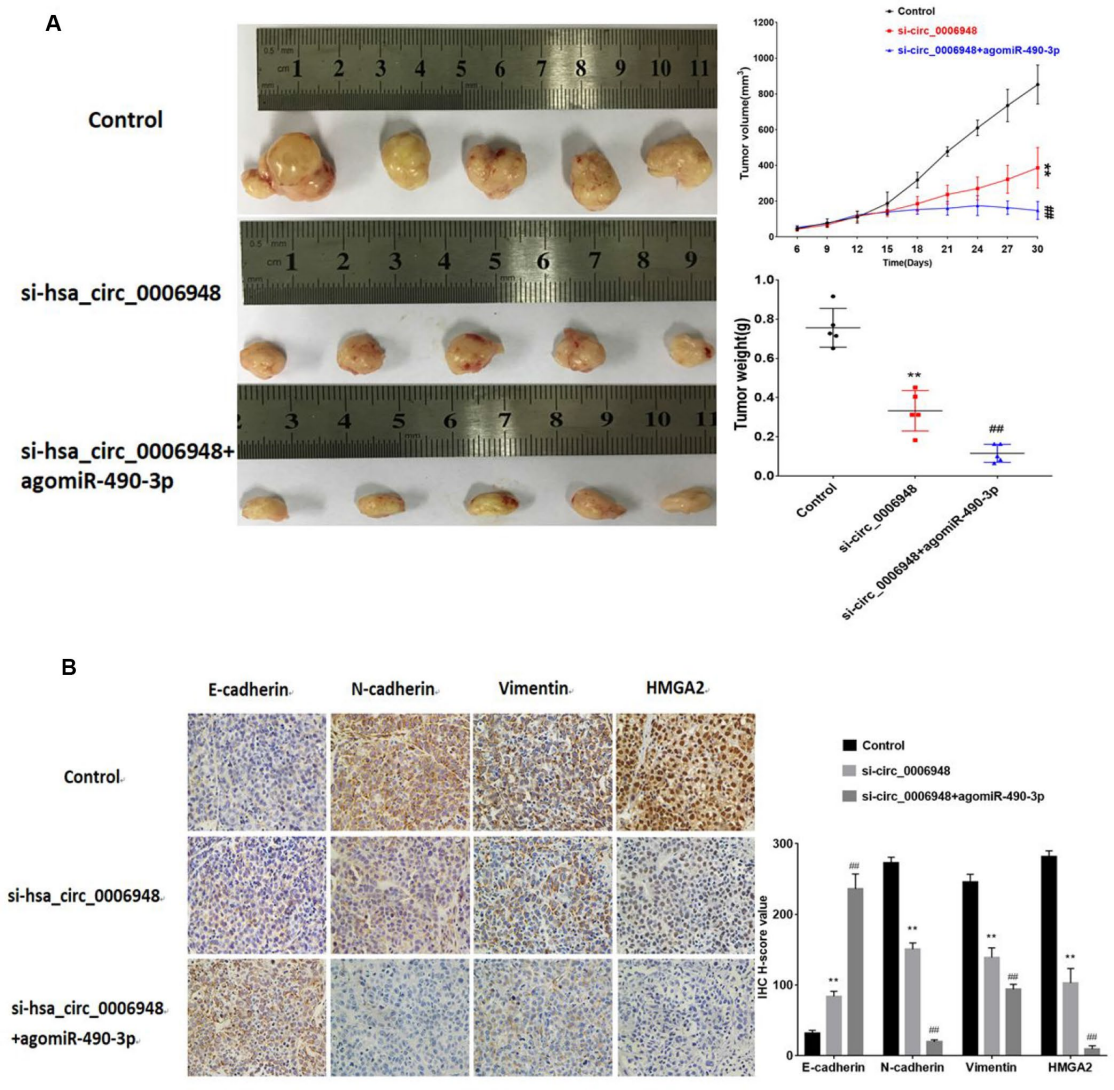
**The images of tumor-bearing nude mice from three treatment groups (n = 5 for each group) on the 30^th^ day.** (**A**) Tumor volumes were monitored with a caliper during the time course of 30 days, and tumor weights were also measured at the end of this study.(**B**) IHC analysis of E-cadherin, N-cadherin, vimentin and HMGA2. **P<0.01 vs control group, ## P<0.01 vs si-hsa_circ_0006948 group.

To further assess whether hsa_circ_0006948 exerts a tumor-promoting effect in vivo, a xenograft mouse model was established by subcutaneously injecting TE-1 cells (n=5 for each group). After 12 days, negative control, si-hsa_circ_0006948 and combined si-hsa_circ_0006948 and agomiR-490-3p were injected intratumorally every two days for two weeks. The results indicated that the tumor weight and growth rates were significantly lower in the si-hsa_circ_0006948 group than in the control group. Importantly, the tumor volume and weight were lower in the combined si-hsa_circ_0006948 and agomiR-490-3p group than in the si-hsa_circ_0006948 group alone. We further used IHC to evaluate the tumor tissues. The IHC results showed that the expression levels of HMGA2, N-cadherin and vimentin were significantly inhibited in the combined si-hsa_circ_0006948 and agomiR-490-3p group compared with those in control group or si- hsa_circ_0006948 group alone, which implied that silencing hsa_circ_0006948 combined with miR-490-3p overexpression exhibits an additive inhibitory effect on ESCC growth in xenograft animal models (Figure 8).

## DISCUSSION

CircRNAs played a crucial role in carcinogenesis and cancer progression, and their high conservation and relative stability are two important properties [20]. Thus, circRNA has become a hot topic in carcinoma research. In recent years, although an increasing number of studies have reported that dysregulated circRNAs were confirmed in various human cancers [10, 21–23], the function of the majority of circRNAs remains unclear. In this study, we identified a novel circular RNA termed hsa_circ_0006948 that was up-regulated in ESCC cells and tissues, and higher expression of hsa_circ_0006948 was associated with poor survival and lymph metastasis. Therefore, hsa_circ_0006948 might be a promising biomarker for the prognosis of ESCC.

At present, the role of EMT in tumor progression has become a hotspot. EMT is a key participant in the biological processes such as the formation, invasion and metastasis of multiple cancers. The invasion and metastasis abilities of tumor cells may become stronger due to intercellular connecting substances loss, intercellular polar complex disappearance and structural changes in extracellular matrices [24]. Therefore, it is important that we identify the molecular mechanisms for circRNA function of EMT in ESCC. In the present study, we screened dysregulated circRNAs through microarray analysis and identified that hsa_circ_0006948 promotes proliferation, migration and invasion in vitro and induces tumor growth in vivo, and hsa_circ_0006948 was further confirmed to be an EMT-related circRNA, suggesting its tumor-aggressive effect.

Due to their posttranscriptional function, circRNAs are considered crucial gene regulators. Because circRNAs contain multiple miRNA-binding sites or miRNA response elements, they have the potential to be miRNA sponges. For circRNAs, acting as a miRNA sponge is the most reported commonly function pattern [25, 26]. In the present study, hsa_circ_0006948 was confirmed to harbor miRNA-binding site predicted by bioinformatics and was located mainly in the cytoplasm. Therefore, we speculated that hsa_circ_0006948 may act as a miRNA sponge in ESCC. Furthermore, the luciferase reporter assay and the RIP assay indicated that hsa_circ_0006948 may bind to miR-490-3p directly. Further “rescue” assays suggested that the tumor suppressive roles of miR-490-3p could be partially reversed by hsa_circ_0006948, suggesting that hsa_circ_0006948 functions as a miR-490-3p sponge in ESCC.

miRNAs have been reported to bind the 3′UTR of target mRNAs and regulate tumor progression. To date, several studies have reported that miR-490-3p is a tumor suppressor in multiple types of cancer. For example, in colorectal cancer, miR-490-3p inhibits cancer progression through activating the Wnt/β-catenin signaling pathway [27]. Importantly, Kang et al. showed that miR-490-3p could bind to the target gene HMGA2 and inhibit EMT in ESCC [19]. Our further studies indicated that upregulation of hsa_circ_0006948 significantly increased the expression of miR-490-3p targeting oncogenes HMGA2. Therefore, we speculated that the regulatory axis hsa_circ_0006948/ miR-490-3p/HMGA2 could be involved in the EMT of ESCC. First, consistent with previous study, our data suggested that upregulating miR-490-3p inhibits ESCC cell proliferation, migration and invasion and induces EMT. Second, HMGA2 was identified as the direct target of miR-490-3p [19], and it could promote EMT in ESCC. Third, the effect of hsa_circ_0006948 on HMGA2 could be partially alleviated by overexpression of miR-490-3p, implying a novel regulatory axis formed by hsa_circ_0006948/miR-490-3p/HMGA2 in ESCC.

Adhesion factor, such as E-cadherin, vimentin and N-cadherin, plays important roles in EMT process. HMGA2 have been reported to regulate E-cadherin, vimentin and N-cadherin [28, 29]. As a nonhistone nuclear-binding protein, HMGA2 is a vital regulator of cell growth and differentiation. Furthermore, HMGA2 was revealed to be a regulator of SNAIL1, a critical EMT molecule, by binding to the promoter directly [30, 31]. Therefore, in the further studies, we found that downregulating hsa_circ_0006948 decreased the expression of N-cadherin and vimentin and increased E-cadherin expression level. In addition, the effects of hsa_circ_0006948 on E-cadherin, vimentin and N-cadherin were reversed by overexpression of miR-490-3p in ESCC cells. Furthermore, knockdown of HMGA2 could reverse hsa_circ_0006948-induced increase on vimentin and N-cadherin expression. The above results indicated that hsa_circ_0006948 regulates EMT-related protein via miR-490-3p/HMGA2. Taken together, the novel regulatory EMT-related axis formed by hsa_circ_0006948/miR-490-3p/HMGA2 may supply the potential therapeutic targets for ESCC.

In summary, hsa_circ_0006948 was up-regulated in ESCC tissues and cells, and we found that increased hsa_circ_0006948 was associated with poor survival in ESCC patients. Furthermore, our data indicated that overexpression of hsa_circ_0006948 promotes the cancer progression and could induce EMT by enhancing HMGA2 by sponging miR-490-3p. Our results suggested that hsa_circ_0006948 could be a vital biomarker for ESCC progression.

## MATERIALS AND METHODS

### Ethical statement and tissue collection

This study was approved by the Ethics Committee of Sun Yat-sen Memorial Hospital. All clinical data were collected after each surgery. The esophageal cancer tissues and adjacent normal samples were obtained from patients who had undergone surgery at the Sun Yat-sen Memorial Hospital between January 1, 2014 and December 31, 2016. These 153 pairs of tumor and adjacent tissue specimens were frozen immediately and stored at −80 °C. After being confirmed by experienced clinical pathologists, the tumor and adjacent normal tissues were subjected to further analysis. We obtained written informed consent from all patients before their participation in this research.

### Microarray data processing

circRNA Microarray (GSE131969), which have been deposited at Gene Expression Omnibus (http://www.ncbi.nlm.nih.gov/geo/) previously, was used for the global profiling of human circRNAs. Differentially expressed circRNAs with statistical significance between the two groups were identified through volcano plot filtering. Differentially expressed circRNAs between the two sample types were identified through fold Change filtering. Distinguishable circRNA expression patterns among the samples were shown through hierarchical clustering.

### Total RNA extraction and qRT-PCR

Total RNA was extracted with RNAiso Plus (TaKaRa Japan) in accordance with the manufacturer’s instructions. The purity and concentration of the total RNA samples were measured with a NanoDrop 2000 (Thermo Scientific, Wilmington, DE, USA). All cDNA was generated with PrimeScript RT Master Mix (TaKaRa, Japan).qRT-PCR for circRNAs was performed on a LightCycler® 96 System (Roche, Switzerland). The relative circRNA expression was calculated using:relative expression=2^-ΔCt^ or relative expression=2^-ΔΔCt^.

### Cell culture

ESCC cells (TE-1, KYSE30, TE-7, TE-13, KYSE150 cell lines) and normal epithelial esophageal cells (HEEC cell line) were purchased from the Shanghai Institutes for Biological Science, China. Briefly, esophageal cells were cultured in DMEM (Gibco; Thermo Fishier Scientific, Suzhou, China) with 10% fetal bovine plasma at 37 °C in a humidified atmosphere with 5% CO_2_.

### Oligonucleotide transfection

Before transfection, TE-1 and KYSE30 cells were seeded in 6-well plates to approximately 60% confluence. siRNA, miRNA mimics and inhibitors were transfected using a lipofectamine RNAiMAX transfection kit (Invitrogen, USA), and overexpression plasmids were transfected using a lipofectamine3000 transfection kit (Invitrogen, USA) following the manufacturer’s instructions.

### Cell proliferation assay

To determine whether hsa_circ_0006948 is involved in ESCC cell proliferation, CCK8 assays were performed. TE-1 and KYSE30 cells transfected with si_circ_0006948 and overexpression vectors were seeded into 96-well plates at 5×10^3^ /well and cultured for 24, 48, 72, 96, 120 and 144 h. Cell proliferation was determined using a Cell Counting Kit-8 (CCK-8; Immuno Way Biotechnology Company Plano, TX, USA). OD450 values were then determined. Three biological repeats were performed for the statistical analysis. We used colony-forming assays to evaluate the clonogenic ability of transfected TE-1 and KYSE30 cells. Cells were seeded into 6-well plates (1000/well) and incubated for approximately 2 weeks. The visible colonies were counted after staining with crystal violet.

### Transwell migration and invasion assays

Transwell assays were used to evaluate the migration and invasion of ESCC cells using transwell chambers (Costar, USA) precoated with or without Matrigel. For the assay, 2×10^5^ cells in serum-free medium were added to the upper chambers (pore size, 8 μm; Corning Inc., Tewksbury, MA, USA). DMEM with 10% fetal bovine serum was added to the lower chambers. After incubation for 24 h, the esophageal cancer cells migrated into the lower chambers were fixed in 4% paraformaldehyde and stained with a crystal violet staining solution. Random fields were digitally imaged and counted.

### RNA fluorescence in situ hybridization

To study the location of hsa_circ_0006948, RNA fluorescence in situ hybridization (RNA-FISH) was performed using a Fluorescent in Situ Hybridization Kit (RiboBio, Guangzhou, China) according to the manufacturer’s guidelines. Cy3-labeled hsa_circ_0006948 probes were measured by the Fluorescent in Situ Hybridization Kit, followed by visualization with confocal microscopy.

### RNA immunoprecipitation

RNA immunoprecipitation (RIP) assays were performed using an anti-AGO2 antibody with the transfection of miR-490-3p mimics (miR-490-3p) or miR-NC in TE-1 and KYSE30 cells to detect hsa_circ_0006948 expression according to qRT-PCR.

### Luciferase reporter assay

Hsa_circ_0006948 sequences (or HMGA2-3’UTR sequences) containing wild-type or mutated miR-490-3p binding sites were synthesized and inserted into luciferase vectors respectively. HEK-293T cells were seeded into 24-well plates at a density of 3 × 10^4^ cells per well. After co-transfection with miR-490-3p mimics and constructed luciferase plasmids for 48 h, we measured luciferase activity using a dual-luciferase reporter assay system (Promega, USA) according to the manufacturer′s protocol. Relative luciferase activity was normalized to the Renilla luciferase internal control. Three independent experiments were performed in triplicate.

### Western blot analysis

Proteins were extracted using RIPA buffer (CWBIO, China). After being electrophoresed by SDS-PAGE, the samples were transferred to nitrocellulose membranes and incubated with primary antibodies specific for E-cadherin (diluted 1:1000, Proteintech, USA), vimentin (diluted 1:1000, ABclonal, China), N-cadherin (diluted 1:1000, ABclonal, China), HMGA2 (diluted 1:1000, ABclonal, China) and GAPDH (diluted 1:1000, ABclonal, China) at 4 °C overnight. Then the membranes were incubated with secondary antibodies at room temperature for 1 h. Signals were detected with images acquisition using Immobilon ECL substrate (Millipore, Germany) and Optimax X-ray Film Processor (Protec, Germany).

### Mouse xenograft model

Ethical approval was obtained from the Institutional Research Ethics Committee of Sun Yat-sen University. All animal care and procedures were performed in accordance with institutional guidelines. To assess the effect of hsa_circ_0006948 on tumor growth in xenograft models, TE-1 cells (5×10^6^/0.2 ml PBS) were injected subcutaneously into the right backs of 4-week-old BALB/c nude mice. After 12 days, the mice were treated with intertumoral injection of negative control, si-hsa_circ_0006948 and combined si-hsa_circ_0006948 and agomiR-490-3p every two days, respectively. The tumors were measured every three days and their volumes were calculated according to the following formula: tumor volume = (length × width^2^)/2. Thirty days later, the mice were sacrificed, and the tumors were excised for further immunohistochemistry. Primary antibodies against E-cadherin (Proteintech, USA), vimentin (ABclonal, China), N-cadherin (ABclonal, China) and HMGA2 (ABclonal, China) were used. We captured images using a Nikon Eclipse 80i system with NIS-Elements software (Nikon, Japan).

### Statistical analysis

The relationship between hsa_circ_0006948 expression and clinicopathological features was analyzed by Chi-square test. Overall survival (OS) was assessed by Kaplan-Meier analysis and compared by log-rank test. Univariate and multivariate Cox proportional hazards regression analyses were used to analyze the independent prognosis factors. ROC curve were drawn to assess the diagnosis value and prognosis value of hsa_circ_0006948. P <0.05 was considered statistically significant. Data analyses were performed using PRISM (GraphPad Software Inc., San Diego, CA, USA), and Stata version 13.1 (StataCorp, College Station, TX).

## Supplementary Material

Supplementary Figure 1
