# Correction for: Hsa_circ_0006948 enhances cancer progression and epithelial-mesenchymal transition through the miR-490-3p/HMGA2 axis in esophageal squamous cell carcinoma

**DOI:** 10.18632/aging.102884

**Published:** 2020-02-23

**Authors:** Zihao Pan, Jiatong Lin, Duoguang Wu, Xiaotian He, Wenjian Wang, Xueting Hu, Lei Zhang, Minghui Wang

**Affiliations:** 1 Guangdong Provincial Key Laboratory of Malignant Tumor Epigenetics and Gene Regulation, Sun Yat-Sen Memorial Hospital, Sun Yat-Sen University, Guangzhou 510120, China; 2 Department of Thoracic surgery, Sun Yat-Sen Memorial Hospital, Sun Yat-Sen University, Guangzhou 510120, China; 3 Department of Biliary-Pancreatic Surgery, The Third Affiliated Hospital, Sun Yat-sen University, Guangzhou 510630, Guangdong Province, China

**Keywords:** correction

Original article: Aging. 2019; 11:11937–11954. PMID: 31881015 PMCID: PMC6949050 https://doi.org/10.18632/aging.102519

**This article has been corrected:** The authors requested the replacement of Figure 1C, Figure 7C and Supplementary Figure 1E incorrectly prepared due to mistake in the organization of this paper. The mistakes of these figures are described below:

**Figure 1C:** The label of vertical coordinate should be “Relative expression of circRNA”.

**Figure 7C:** The transwell assay result for the TE1 transfected with vector+si-HMGA2.

**Supplementary Figure 1E:** The transwell invasion assay result for KYSE30 transfected with si-hsa_circ_0006948.

These corrections do not change the content of the publication.

**Figure 1:**
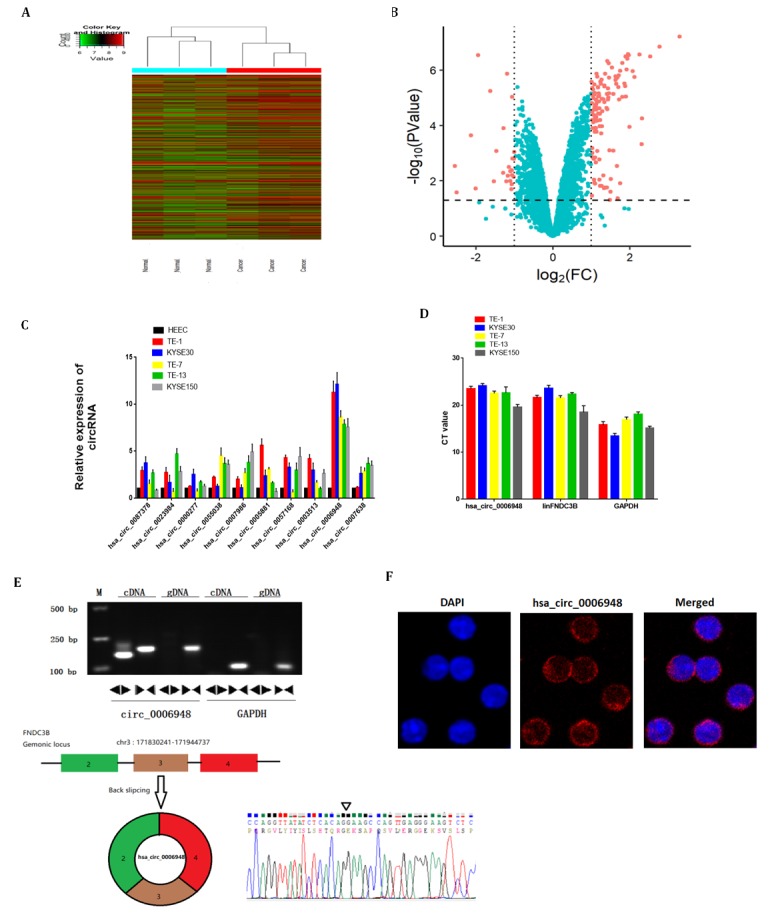
**The identification and characteristics of hsa_circ_0006948 in ESCC cells. **(**A**) Heat map showing the differential expression and hierarchical clustering of circRNAs between ESCC and adjacent normal tissues. (**B**) Volcano plot, x-axis: log2 (fold change); y-axis: -log10 (P-value). The vertical lines correspond to 2.0-fold up and down, and the horizontal line represents a P-value of 0.05. The red points in the plot represent differentially expressed circRNAs with statistical significance. (**C**) The relative hsa_circ_0006948 was significantly high in ESCC cells. (**D**) q RT-PCR analyses of expression of hsa_circ_0006948, linFNDC3B and GAPDH in various ESCC cell lines. Y-axis is the raw CT value. (**E**) Above: Divergent primers detected circular RNAs in cDNA but not gDNA. Below: Three exons form hsa_circ_0006948 by back splicing from chromosomal region and Sanger sequencing of hsa_circ_0006948 showed the back-splice junction (∇).** (F) **Fluorescence in situ hybridization assay was conducted to determine the subcellular localization of hsa_circ_0006948.

**Figure 7:**
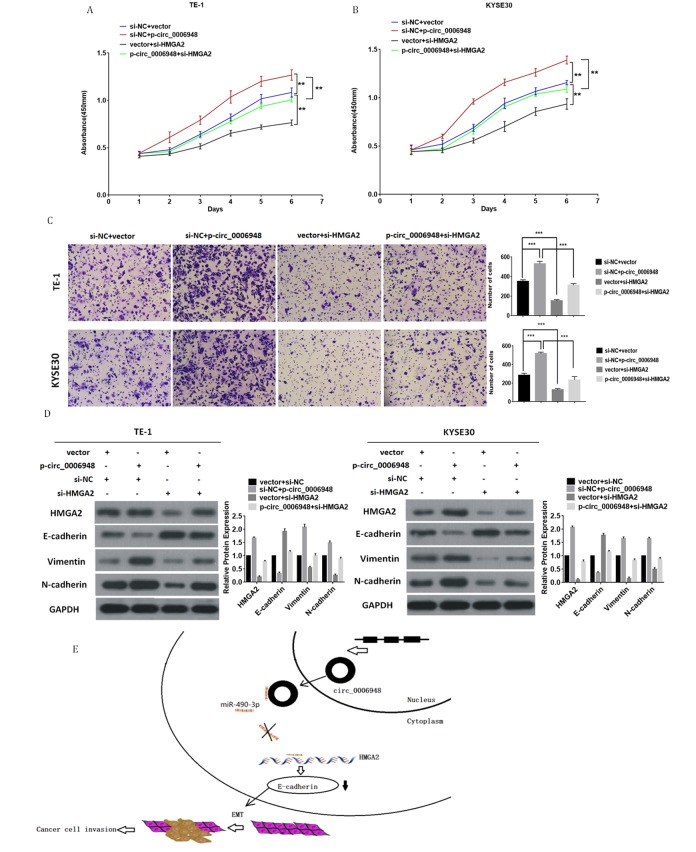
**Knockdown of HMGA2 abolishes the oncogenic effect induced by hsa_circ_0006948 in ESCC. **(**A **and** B**) The cell proliferation was measured by CCK8 assays. (**C**) The invasion ability was evaluated by transwell Matrigel invasion assays. (**D**) The upregulation of vimentin, N-cadherin. HMGA2 and the downregulation of E-cadherin in TE-1 and KYSE30 cells transfected with hsa_ciic_0006948 overexpression plasmid were abolished by knockdown of HMGA2 as detected by Western blot analysis. (**E**) A mechanistic model: hsa_circ_0006948 functions as a miR-490-3p sponge and regulates HMGA2 through inhibiting miR-490-3p activity in ESCC cells' EMT. *P<0.05, **P<0.01, ***P<0.001.

**Supplementary Figure 1:**
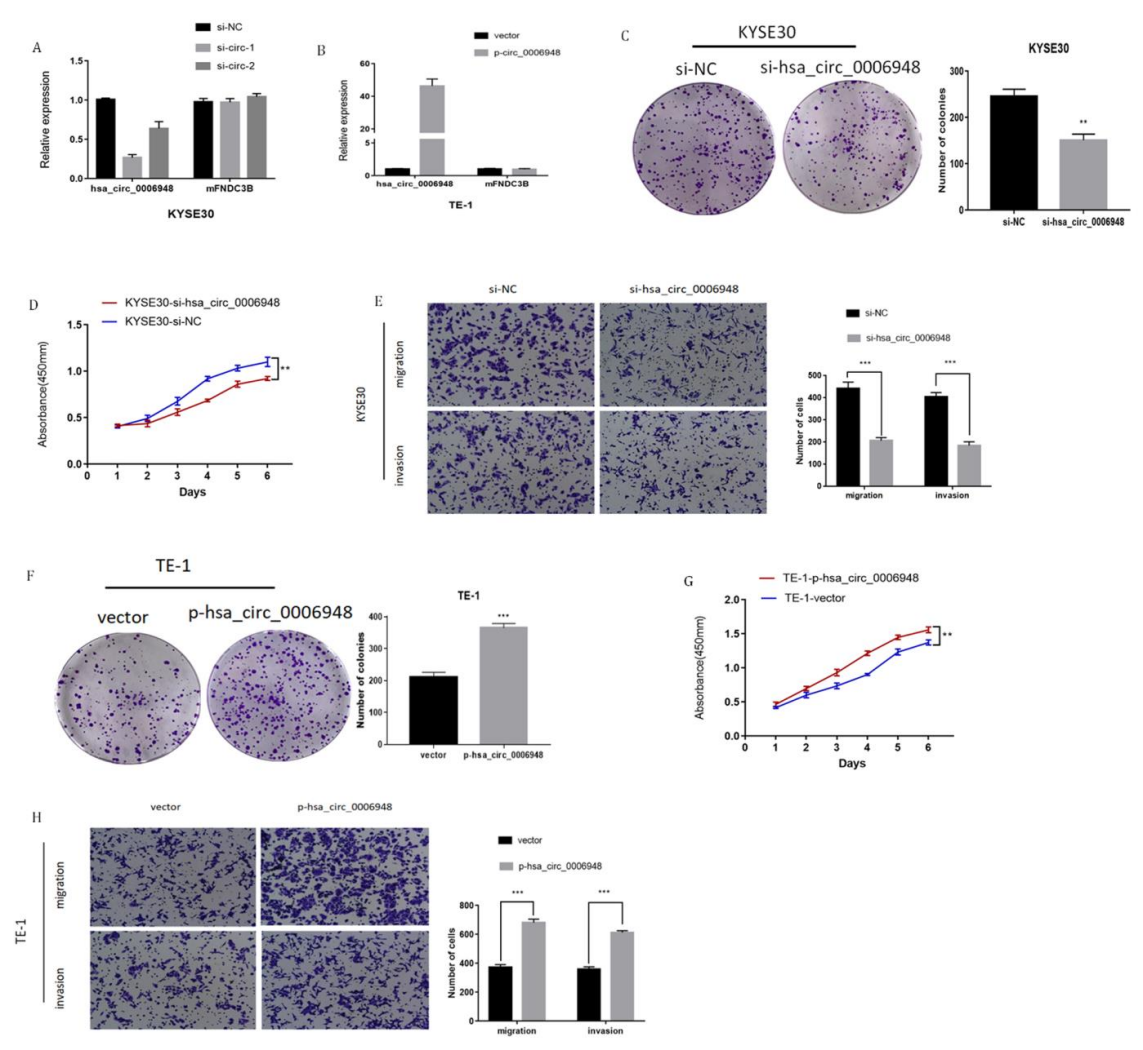
**The function of hsa_circ_0006948 in ESCC cells. **(**A**) Expression of hsa_circ_0006948 and FNDC3B mRNA in KYSE30 cells transfected with siRNAs and (**B**) TE-1 cells overexpressing hsa_circ_0006948. (**C **and** D**) The effect of hsa_circ_0006948 on cell proliferation in vitro using colony formation assay and CCK8 assay after knocking down hsa_circ_0006948 in KYSE30. (**E**) Cell migration and invasion abilities were assessed by transwell assay after knocking down hsa_circ_0006948 in KYSE30 cells. (**F **and** G**) The effect of hsa_circ_0006948 on cell proliferation in vitro using colony formation assay and CCK8 assay after overexpressing hsa_circ_0006948 in TE-1 cells. (**H**) Cell migration and invasion abilities were assessed by transwell assay after overexpressing hsa_circ_0006948 in TE-1 cells.* P<0.05, **P<0.01, ***P<0.001.

