# Supplementary material for: Hsa_circ_0006948 enhances cancer progression and epithelial-mesenchymal transition through the miR-490-3p/HMGA2 axis in esophageal squamous cell carcinoma
Source: Aging (Albany NY). 2019 Dec 26;11(24):11937–54. doi: 10.18632/aging.102519 (PMC6949050; doi:10.18632/aging.102519)
Supplement: Supplementary Figure 1 [file aging-11-102519-s001..pdf]

SUPPLEMENTARY FIGURES

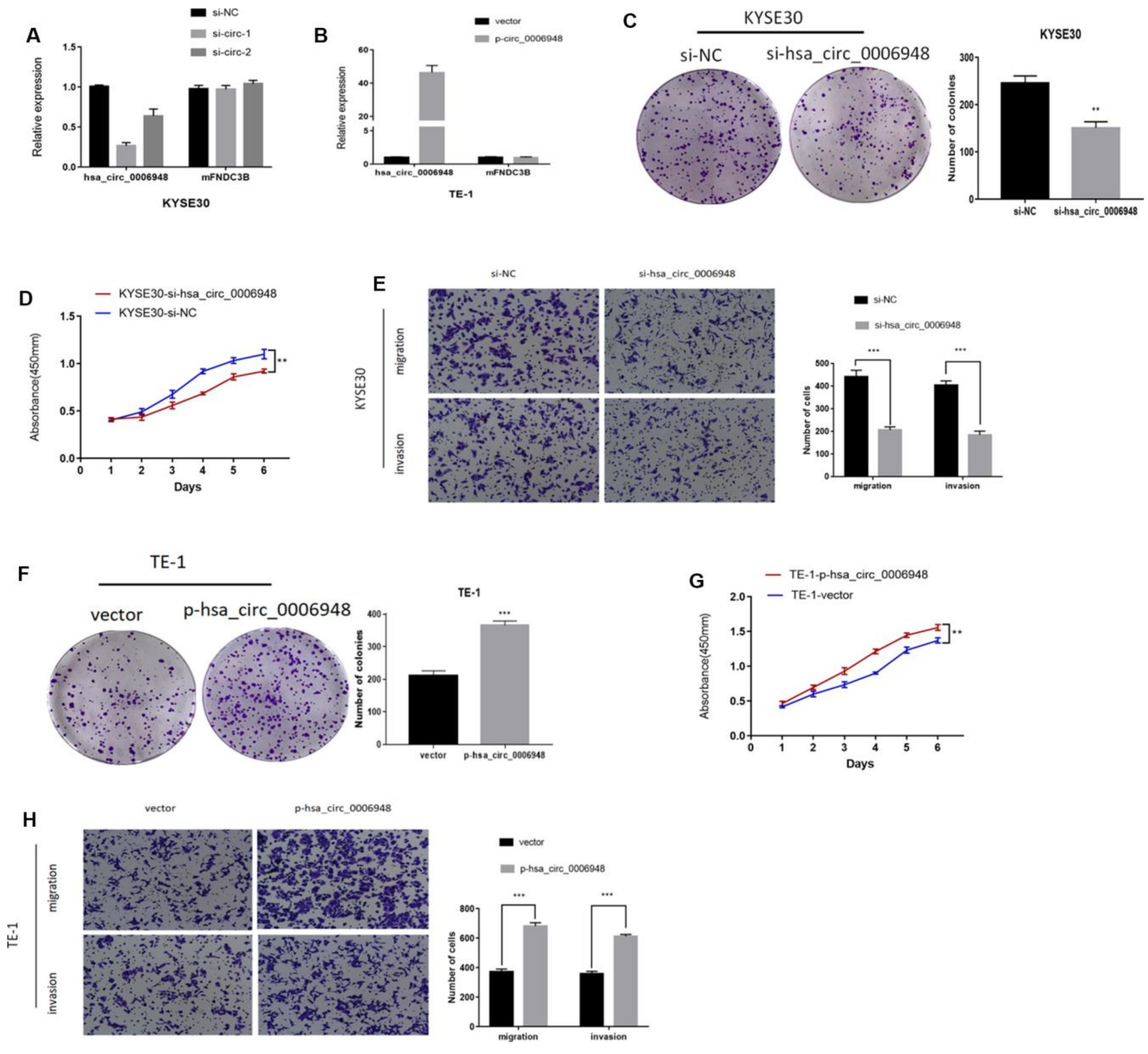

Supplement Figure 1. The function of hsa_circ_0006948 in ESCC cells. (**A**) Expression of hsa_circ_0006948 and FNDC3B mRNA in KYSE30 cells transfected with siRNAs and (**B**) TE-1 cells overexpressing hsa_circ_0006948. (**C** and **D**) The effect of hsa_circ_0006948 on cell proliferation in vitro using colony formation assay and CCK8 assay after knocking down hsa_circ_0006948 in KYSE30. (**E**) Cell migration and invasion abilities were assessed by transwell assay after knocking down hsa_circ_0006948 in KYSE30 cells. (**F** and **G**) The effect of hsa_circ_0006948 on cell proliferation in vitro using colony formation assay and CCK8 assay after overexpressing hsa_circ_0006948 in TE-1 cells. (**H**) Cell migration and invasion abilities were assessed by transwell assay after overexpressing hsa_circ_0006948 in TE-1 cells.* P<0.05, **P<0.01, ***P<0.001.
